# Case Report: Life-threatening Kasabach–Merritt phenomenon in a 2-month-old child

**DOI:** 10.3389/fped.2025.1690450

**Published:** 2025-10-31

**Authors:** Ashley V. Wong Grossman, Juan Pablo Forero, Jeffrey S. Yu, Megan M. Gilbert, Kanwaljeet J. S. Anand

**Affiliations:** ^1^ Department of Anesthesiology and Perioperative Medicine, Mayo Clinic, Rochester, MN, United States; ^2^ Department of Pediatrics, Stanford University School of Medicine, Stanford, CA, United States; ^3^ Department of Pediatrics, Hematology & Oncology, Stanford University School of Medicine, Stanford, CA, United States

**Keywords:** infant, lymphatic disorder, tumor, coagulopathic bleeding, vascular malformation

## Abstract

We present a rare case of a 2-month-old girl, born at term, who was admitted to the pediatric intensive care unit for fussiness, increasing abdominal distension, and intermittent diarrhea for one week. She was found to be hypertensive, coagulopathic, and suffering from unrelenting ascites. Over the course of the following month, her symptoms became life-threatening, and she was intubated, sedated, and paralyzed. She underwent multiple diagnostic and therapeutic procedures, including exploratory laparotomy, MRI, colonoscopy, and multiple peritoneal drains. After several weeks, the medical teams reached a consensus diagnosis of a rare and complex vascular anomaly characterized by a life-threatening Kasabach–Merritt Phenomenon (KMP). KMP is known to occur in association with several anomalies, including kaposiform hemangioendothelioma and kaposiform lymphangiomatosis. Life-saving therapies were initiated, including methylprednisolone and sirolimus. The patient was ultimately discharged and sent home with her parents. To our knowledge, this is the only reported case describing this constellation of symptoms in a critically ill infant. Multidisciplinary cooperation was key in saving the patient's life. Critical care providers should consider vascular anomalies in patients with similar presentations.

## Introduction

Kasabach–Merritt phenomenon (KMP) is an extremely rare clinical phenomenon characterized by thrombocytopenia, hemolytic anemia, and life-threatening consumptive coagulopathy in the setting of a rapidly enlarging vascular tumor. Its incidence is estimated to affect .07 per 100,000 children per year (1). Left untreated, KMP has a mortality rate of 10%–30% (2). KMP is most frequently seen in vascular tumors, including tufted angioma (TA) and kaposiform hemangioendothelioma (KHE) (3), but can also be seen in some complex vascular anomalies such as kaposiform lymphangiomatosis (KLA) (4).

All ethnicities and genders appear to be equally affected by KMP. Associated with vascular conditions, it often presents in infancy, with one case series showing a mean age of diagnosis of 2 months (5).

The diagnosis can be made clinically if the vascular lesion is discrete and visible, which is not always the case. Ideally, a biopsy of a lesion is obtained for confirmatory staining. Often, such a biopsy is not possible because of the concomitant KMP and risk of bleeding. Affected areas often include the face, extremities, trunk, and retroperitoneum. With appropriate treatment, most patients see significant improvement within days to weeks.

Our case report aims to discuss the broad differential diagnosis and diagnostic challenges encountered in the unique presentation of our patient, as well as highlight the nuances across the spectrum of conditions such as KHE and KLA.

## Case description

### Investigations

A 2-month-old girl was brought to the emergency department by her parents for fussiness, increasing abdominal distension, and intermittent diarrhea for one week. The patient was born at term following an uncomplicated pregnancy. The patient was discharged following a normal newborn course and had recently received her 2-month vaccinations. Her physical examination was notable for infant irritability and she presented with a soft but markedly distended abdomen with diffuse tenderness to palpation without rebound or guarding. She had prominent ecchymosis on her labial folds. Her vital signs were notable for tachycardia (heart rate 140 s) and hypertension (blood pressure 130 s/70 s). The results of her cardiac, respiratory, and neurologic tests were normal. Initial laboratory studies (Table 1) revealed severe anemia, thrombocytopenia, and elevated reticulocyte levels. Coagulation studies revealed prolonged prothrombin and thrombin times, low fibrinogen, and an elevated D-dimer, suggesting disseminated intravascular coagulation (DIC). After receiving 15 mL/kg of packed red blood cells and 15 mL/kg of platelets, she was admitted to the PICU for continued workup and management.

**Table 1:** Laboratory values on admission to the PICU.

Test	Results	Units
Complete blood count		
White blood cells	10.3	×10^3^ mcL
Hemoglobin	4.9	g/dL
Hematocrit	15.8	%
Platelet count	7	k/μL
Segmented neutrophils	31	%
Lymphocytes	54	%
Monocytes	12	%
Bands	3	%
Reticulocytes	21.1	% (0.32 m/μL)
Coagulation studies		
Prothrombin time	18.2	s
Partial thromboplastin time	35.3	s
Thrombin time	21.2	s
Fibrinogen	68	mg/dL
D-dimer	>20.0	μg/mL FEU
ADAMTS activity	57	%
Basic metabolic panel		
Serum sodium	126	mmol/L
Serum potassium	5.7	mmol/L
Serum chloride	97	mmol/L
Carbon dioxide	21	mmol/L
Urea nitrogen	18	mg/dL
Creatinine	0.31	mg/dL
Glucose	108	mg/dL

While admitted, extensive investigations ensued. Subspecialists in hematology/oncology, gastroenterology, pediatric surgery, and infectious disease were consulted. The patient’s initial workup included an abdominal ultrasound, an abdominal computed tomographic (CT) scan, a CT angiogram (CTA) of the abdomen and pelvis, and a magnetic resonance imaging (MRI) of the abdomen. The only noteworthy findings across these studies were the presence of large-volume abdominal and pelvic ascites, with marked pancolonic and rectal wall thickening, favored to represent colitis. No mass or liver abnormality was identified.

The results of additional diagnostic studies—including a skeletal survey, dilated ophthalmologic examination, head ultrasound, transthoracic echocardiogram, and bone marrow biopsy—were either normal or non-revelatory. A colonoscopy was performed at the patient’s bedside with a limited tissue sample. Pathology reported colonic mucosa with focal vascular congestion and rare cytomegalovirus (CMV) inclusions. The results of liver function tests were normal, as also her ammonia levels. Inflammatory markers showed normal ESR and CRP with ferritin at 495 ng/mL. The infectious workup—comprising blood, urine, and stool cultures; bacterial and AFB cultures from peritoneal fluid; gastrointestinal polymerase chain reaction (PCR) studies; and an Epstein–Barr Virus (EBV) test—demonstrated negative results. The result of a PCR test of the plasma, peritoneal fluid, bone marrow, and urine was positive for CMV. Because of concerns that she may develop a culture-negative infection and carry a significant risk arising out of non-administration of antimicrobials given her critical illness and overall instability, the patient was maintained on broad-spectrum antimicrobials, including ganciclovir. A bone marrow biopsy and aspirate did not reveal a lymphoid malignancy or solid tumor metastases.

Unfortunately, the patient continued to clinically deteriorate during the period of her hospitalization. She received supportive care and analgesia with continuous opioid infusion. However, she remained persistently hypertensive despite the initiation of adequate pain control measures and was therefore placed on sodium nitroprusside infusion. Her ascites continued to accumulate, and loculations developed, necessitating peritoneal drains in all four abdominal quadrants. She had an acute increase in bladder pressure of >50 mmHg (normal <10 mmHg), increased abdominal distension, and continued daily requirements for blood products.

For optimal medical management of her abdominal compartment syndrome, the patient was intubated, sedated, and paralyzed; however, there was no significant reduction in abdominal pressure. She was taken emergently for a decompressive laparotomy. During the performance of this procedure, the entire colon was found to be extremely thickened, with the consistency of a rubber hose, according to the surgeons. The bowel appeared grayish-purple, consistent with likely necrosis, but atypical in the extent of its thickness. The bowel wall also had an unusual microbubble-like appearance, suggesting an infiltrative process. The retroperitoneum and pelvic preperitoneal tissue were significantly firm and hemorrhagic, which appeared to contribute most to increased pressure within the peritoneal cavity.

Following these intraoperative findings, concerns about the patient developing a complex vascular anomaly in the retroperitoneum as an etiology of her unrelenting ascites increased significantly. Clinical suspicion was highest for lesions that present with KMP, such as KHE or KLA. A review of prior imaging revealed small bubble-like areas within the retroperitoneum that were previously thought to be hematoma or fat, but upon further analysis were thought to demonstrate hyperattenuation of the right and left adrenal glands, suggestive of retroperitoneal hematoma or vascular malformation. Her presentation and clinical course were discussed with vascular anomaly experts across the country, who agreed that this was clinically consistent with a presentation of KHE or KLA, although it was atypical due to the fact that the retroperitoneum was the area that was predominantly affected. Although KHE and KLA typically display avid enhancement on T2-weighted imaging, the lack of MRI enhancement in our patient did not rule out these etiologies. With no other alternative diagnostic leads, the patient's declining clinical status, and the life-threatening risks of a retroperitoneal biopsy, the benefit of empiric therapy with methylprednisolone and sirolimus for KHE or KLA was determined to outweigh the risks of treatment.

**Figure 1:**
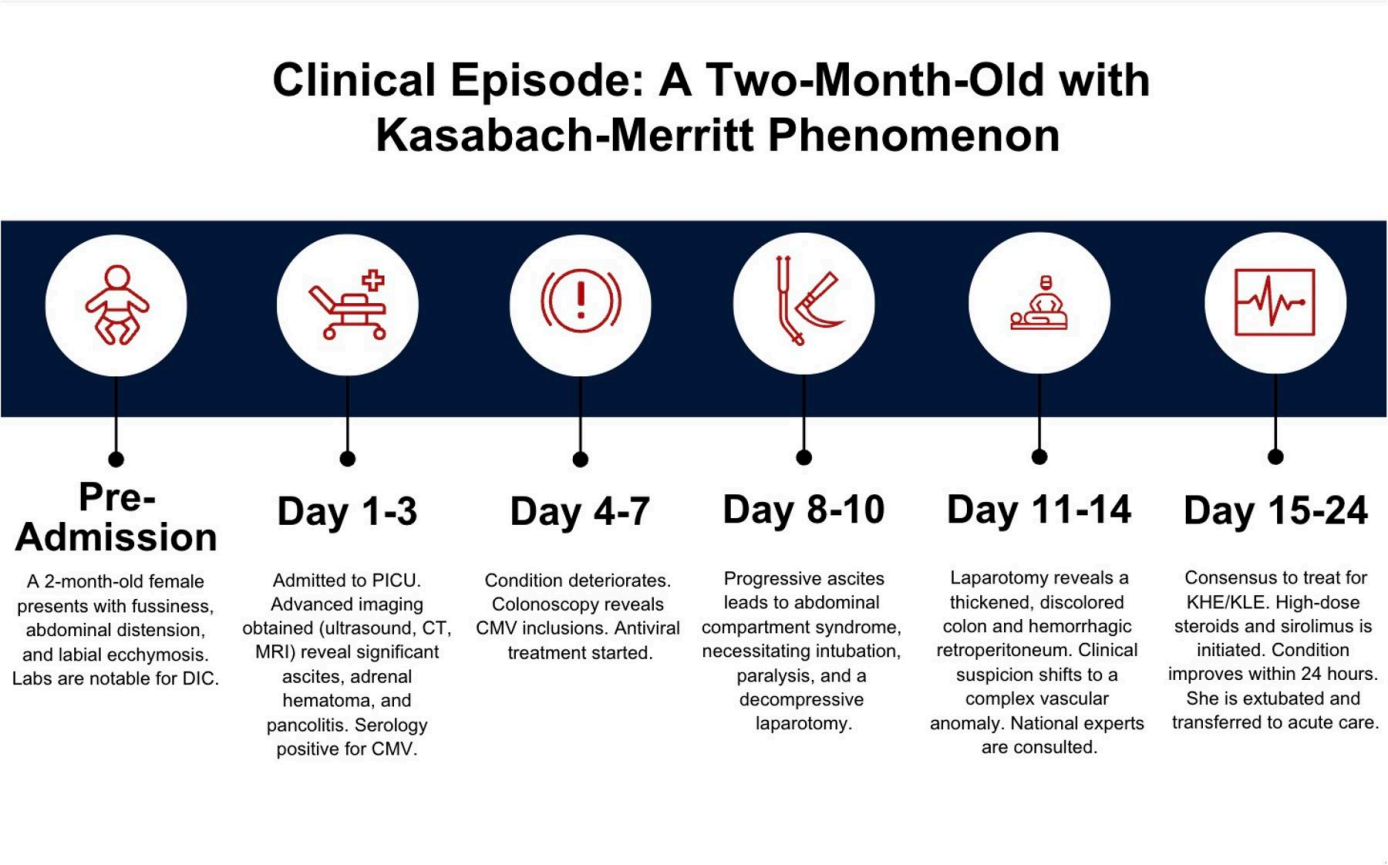
Timeline of clinical progression.

This moribund patient was started on high-dose intravenous methylprednisolone and sirolimus via a nasogastric tube. Within 24 h, her blood product requirements declined and her clinical condition stabilized. Her irritability slowly began to subside, her abdominal condition showed gradual improvement (as revealed by the test result), and coagulopathic bleeding slowly stopped (as revealed by coagulation studies) (Timeline, Figure 1).

**Figure 2:**
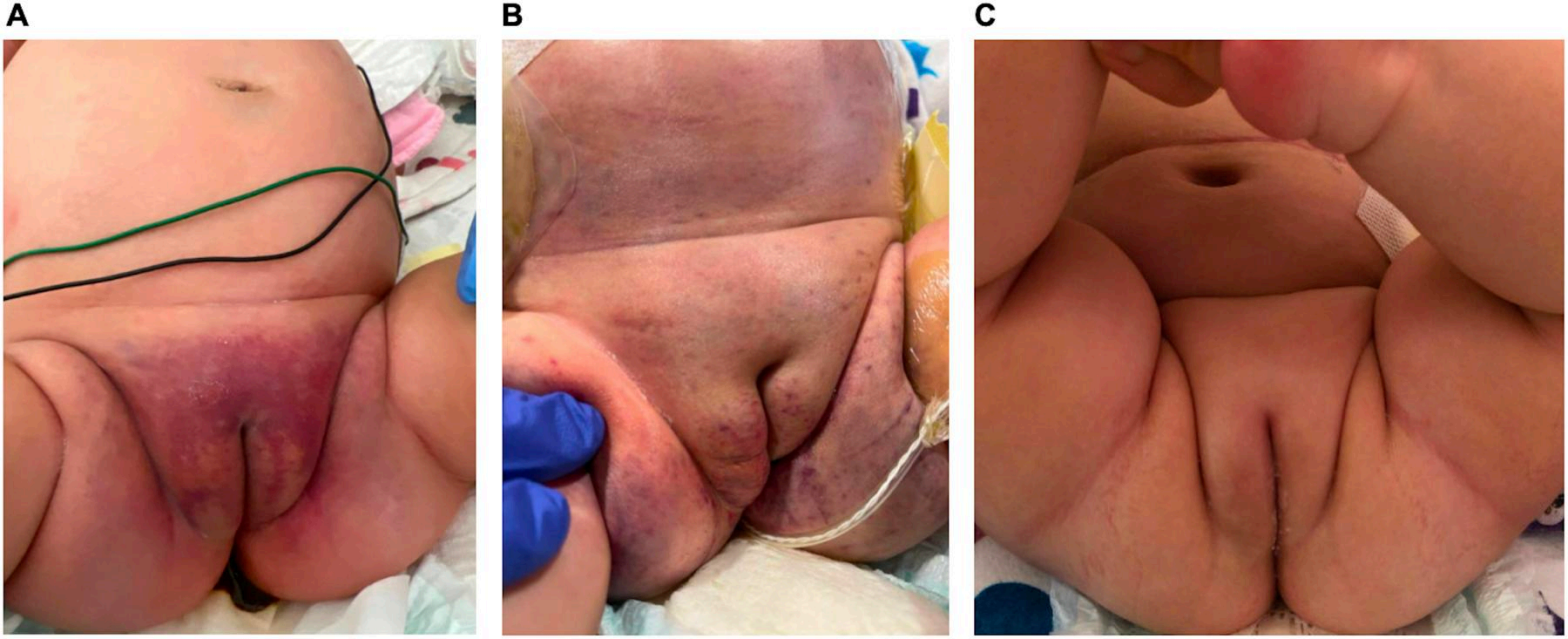
Interval progression of labial ecchymosis. **(A)** Initial presentation of petechiae of the groin and medial thighs coalescing into a larger purpuric patch, **(B)** after starting empiric methylprednisolone and sirolimus with interval improvement but continued edema of the right labia, **(C)** full resolution. Dates: **(A)** 18 April 2023; **(B)** 11 May 2023; **(C)** 16 August 2023.

The patient's ascites began to decline, labial ecchymosis dramatically reduced (Figure 2), and her abdominal incision was closed 2 weeks following the open laparotomy procedure. As her clinical status improved, she was extubated and transferred to acute pediatric care. Her hospitalization was prolonged because of severe feeding intolerance and TPN dependence in the setting of an ileostomy.

### Diagnostic assessment

#### Diagnostic challenges

Our teams encountered several diagnostic challenges. In particular, the coagulopathy associated with KMP carried the risk of life-threatening bleeding with any procedure. Also, this phenomenon made obtaining adequate tissue samples almost impossible. In addition, the small size of our young 2-month-old patient size rendered the carrying out of invasive procedures more technically difficult. Over time, her tenuous hemodynamics, respiratory compromise, and overall critical illness made any diagnostic workup highly risky. Therefore, we took utmost care to balance the benefit of diagnostic studies against the extremely high risk of bleeding and the need for transporting the patient from the intensive care unit. During our patient's most tenuous times, we found that she was unable to be transported, which further limited us to the care that we could provide at her bedside in the unit.

#### Differential diagnosis

An exhaustive multidisciplinary differential was compiled for this patient. Her constellation of findings included ascites, coagulopathy, and hypertension, which suggested a myriad of potential diagnoses.

From a gastrointestinal standpoint, the etiology of ascites in children varies depending on the age group. The most common causes across all age groups are intrahepatic pathologies, such as biliary atresia, followed by hepatic infections, neoplasm, congestive heart failure, and idiopathic causes (6). In such patients, diagnosis frequently follows an often-straightforward workup, usually including a liver biopsy. In our patient, a liver biopsy was performed intraoperatively, which yielded normal results. Occasionally, ascites may represent a finding of rare pathologies. In this case, hepatoblastoma, neuroblastoma, or other solid malignancies are essentially ruled out by early imaging.

From an infectious disease perspective, the CMV PCR was the only positive finding in our patient. CMV colitis, as the etiology of her clinical findings, would not be expected because it normally affects immunocompromised hosts. In addition, hypertension, diffuse ascites, and significant coagulopathy are not typically associated with CMV. While our patient’s limited colonic biopsy revealed rare CMV inclusions, there was no inflammatory infiltrate. In the absence of other end-organ damage, she was diagnosed with a primary CMV infection. Ganciclovir was continued, while her workup remained in a state of progress.

Hematologic and oncologic considerations for our patient were extensive. Given her ongoing anemia, thrombocytopenia, and coagulopathy, the underlying etiologies of DIC such as sepsis, malignancy, trauma, or liver disease were strongly considered. In infants, congenital homozygous deficiency of protein C or S can lead to purpura fulminans and associated DIC. However, in our patient, normal protein C and S activity ruled out these conditions. Solid tumors, of which neuroblastoma, hepatoblastoma, and Wilms’ tumor are the most common (7), present at a higher frequency in infants younger than one year of age when compared with leukemia. In our patient, a negative bone marrow biopsy and aspirate, combined with imaging negative for a mass, made a diagnosis of malignancy unlikely.

Etiologies of thrombocytopenia and microangiopathic anemia (MAHA) were also considered in our patient. Thrombotic thrombocytopenic purpura was ruled out early because she presented with an adequate ADAMTS13 activity level of 57%, with stable serial levels. Other etiologies of MAHA and thrombocytopenia, such as hemolytic uremic syndrome (HUS) and complement-mediated thrombotic microangiopathy (cTMA), were also taken into account. With a negative gastrointestinal pathogen PCR, resolved diarrhea, and a normal creatinine level, HUS was unlikely. The onset of symptoms related to MAHA and thrombocytopenia due to cTMA, a hereditary deficiency in proteins that regulate the alternative complement pathway, can be sudden and frequently follow an infectious etiology in children (8). With ongoing hypertension, cTMA was a concern in our patient, but this did not explain the significant bowel findings on imaging and the unrelenting ascites. A rapid cTMA genetic panel was performed, but it yielded an equivocal result with a variant of unknown significance. Then, because of the worsening of her condition, the patient was empirically given a dose of eculizumab, but there was no discernible clinical change or a rise in her platelet count; therefore, this diagnosis was also considered improbable.

Another etiology of DIC that has become most prominent is Kasabach–Merritt phenomenon (KMP). KMP is a life-threatening coagulopathy that presents with severe thrombocytopenia, hypofibrinogenemia, and the consumption of additional clotting factors in conjunction with a vascular tumor such as a tufted angioma (TA) or kaposiform hemangioendothelioma (KHE) (3). This condition is often visualized through a rapidly enlarging lesion or tumor. However, in our patient, imaging was negative for an enhancing mass or lesion, and her labial ecchymosis reduced with the transfusion of platelets and fresh frozen plasma (Figure 2).

**Figure 3:**
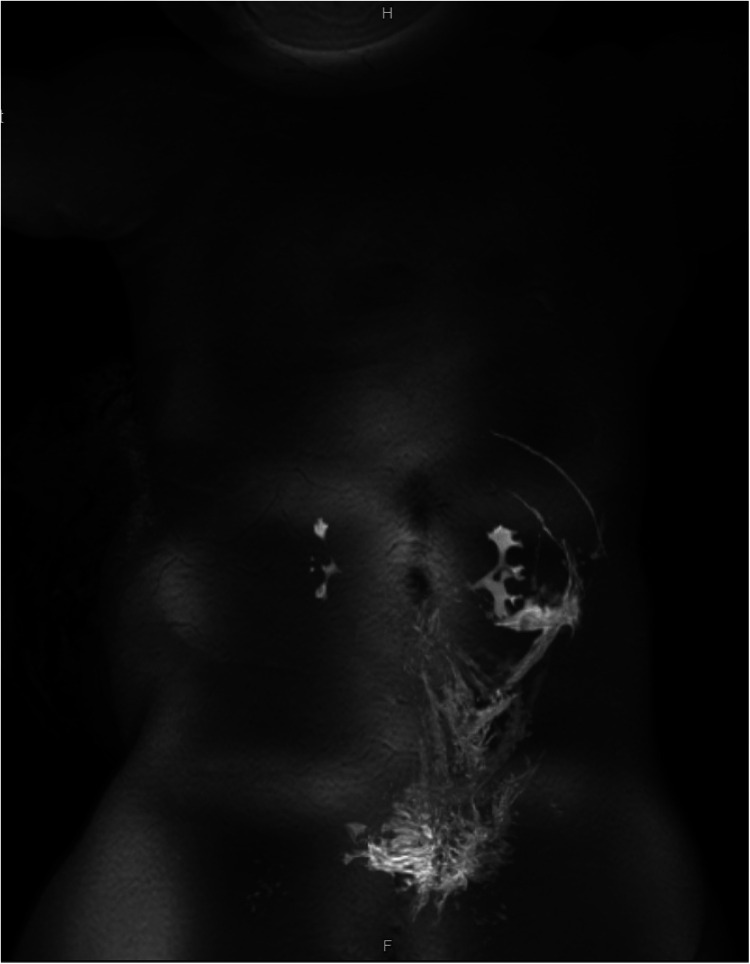
MRI lymphangiogram. Majority of contrast spreads along the left retroperitoneal and perirenal space.

Following worrisome findings in the retroperitoneum during her exploratory laparotomy, the patient’s limited colonic biopsy sample was restained to further evaluate it for complex vascular malformations. Endothelial cells within the areas of vascular congestion were positive for lymphatic markers (D2-40 and Prox-1) with variable CD34 expression, consistent with lymphatic endothelial cells in a vascular malformation, such as KHE, KLA, or multifocal lymphangioendotheliomatosis with thrombocytopenia (MLT). Angiopoietin-2, a protein with roles in angiogenesis and vascular homeostasis, can be elevated in both KLA and KHE (9). Our patient's serum angiopoietin-2 level was then tested and found to be 13,900 pg/mL, exceeding three times the upper limit of normal (range 1,434–4,141 pg/mL), further supporting a diagnosis of KHE or KLA. Eventually, an MRI lymphangiogram (Figure 3) was obtained following the patient's initial hospital discharge. Most of the lymphatic contrast injected into an inguinal lymph node spread along diffuse infiltrative soft tissue within the left retroperitoneum, all consistent with KHE or KLA.

#### Treatment

The treatment approach for KHE or KLA is not standardized and must be tailored to the location, size, and associated symptoms of the tumor (10). Surgical resection may be curative for localized KHE, but this is often not possible because of infiltration across tissue planes, as was the case in our patient. Multiple modes of medical management have been described for KHE, including corticosteroids, single and combination chemotherapy agents, radiation, topical therapies, vascular embolization, and sclerotherapy (10). Medical therapy has been shown to lead to remission (11, 12).

First-line therapy for KHE with KMP previously included steroids and vincristine following expert consensus recommendations in 2013 (13). Because the mammalian target of rapamycin (mTOR) is overexpressed in cutaneous vascular malformations (14), sirolimus, an mTOR inhibitor, was tested in an off-label trial for both KLA and KHE with varied effects. A Phase II trial demonstrated the efficacy and safety of sirolimus in a group of patients (*n* = 61) with complicated vascular anomalies, which included patients with KLA (*n* = 7) and KHE (*n* = 13). Although there were varying degrees of response and some patients displayed progressive disease, the majority of patients with complicated vascular anomalies demonstrated beneficial overall responses (15). Sirolimus has a manageable a side-effect profile that includes mucositis, hypertension, hypertriglyceridemia, and hypercholesterolemia. Importantly, as our patient had extensive bowel involvement and had undergone ileostomy, sirolimus can be given orally and has a long half-life (60 h). Vascular experts nationwide routinely utilize sirolimus, with or without steroids, as the first-line medical therapy for KHE with KMP.

In comparison with KHE, KLA appears to be more refractory to medical management and is associated with lower overall survival (16). Combination medical modalities such as steroids, sirolimus, and vincristine are used in the treatment of KLA; however, many patients show a limited response to therapies (4, 17). Vincristine was an attractive option for initial therapy in our patient given its utilization in the treatment of both KHE and KLA; however, its effect of slowing gut motility in this patient who had an open abdomen and known ileus was deemed high risk for bowel obstruction. Vincristine was later added our patient’s outpatient treatment regimen following improvement in her bowel motility.

#### Follow-up and outcome

Our patient was ultimately discharged and sent home with her parents after a prolonged hospitalization because of feeding intolerance and TPN dependence in the setting of an ileostomy. Our colleagues in the hematology and oncology departments continue to follow her up closely. She has been successfully weaned off steroids and remains on sirolimus monotherapy. Ileostomy takedown and reanastomosis has been achieved nearly 2 years after her initial presentation.

## Discussion

KHE is a rare vascular neoplasm that was first described by Zukerberg et al. (18). It has an annual incidence rate of 0.071 per 100,000 children (5, 19). KHE is an aggressive, endothelial-derived tumor that typically affects the face, neck, extremities, or thorax, and cutaneous involvement is seen in over 90% of cases (11, 12). Retroperitoneal involvement is extremely rare. Given its rarity, large-scale studies of KHE have not been performed as yet, and its pathogenesis remains unclear. KHE is almost exclusively seen in the pediatric population, with 90% of cases presenting before 1 year of age and up to 50% of superficial lesions being visible at birth (20). Early tumorigenesis is likely, given its presentation in infancy, as well as prenatally reported findings (21). Over 70% of KHE cases present with KMP, and the risk is highest for large congenital lesions (5, 20).

KLA can be difficult to differentiate from KHE because of the overlap of clinical symptoms, accompanying thrombocytopenia and coagulopathy, and shared histopathologic findings (16). First described in 2014 and initially thought to be an aggressive generalized lymphatic anomaly, KLA is a distinct complex vascular anomaly composed of malformed lymphatic channels with clusters of spindle-shaped lymphatic endothelial cells. Histologically, both KLA and KHE are characterized by diffuse lymphatic malformation, but spindle cells are found in focal clusters in KLA and more diffusely in KHE (5). KLA can be multifocal or diffuse, with many cases involving the thoracic cavity, including the lungs and mediastinum, often presenting with respiratory symptoms and pleural effusions (4). KLA can also be seen within the abdomen, including inside the spleen, bone, liver, and soft tissue. Somatic activating NRAS mutations have been characterized in the majority of KLAs (22).

In addition to the disease-specific considerations that we encountered in our patient in this study, the complexity of this case also highlighted the strengths and limitations of a large, multidisciplinary assessment. Our patient benefited from the vast experience of specialists from all relevant fields, who provided expert guidance and recommendations. One limitation that we faced in this study was that the results of her workup did not match the multiple aspects of her complicated clinical picture. When first-line diagnostic modalities were unrevealing or inconclusive, we as a team were compelled to collectively pivot our thinking. The act of working together took time. Diagnostic testing and procedures needed to be prioritized. Although efficiently done, it added a layer of complexity to the care of this patient. A second, significant limitation of this study was our inability to safely obtain a larger tissue biopsy of the colon because of the coagulopathy of KMP. A biopsy of the retroperitoneum would have been technically unfeasible. Given the poor safety profile of biopsy for this patient, more tissue could not be obtained.

Between KHE and KLA, the presumed diagnosis for our patient was KLA because of the diffusely infiltrative nature of her lesion, the immunohistostaining that is consistent with KLA and KHE, and the extraordinarily elevated angiotensin-2 levels in KLA and KHE. Thankfully, her treatment remains the same for both KLA and KHE, and no further clinical differentiation is necessary at this point of time. Currently, our patient's ascites is resolved; she has remained dependent on parenteral nutrition for over a year because of continued feeding intolerance. She has tolerated increasing volumes by mouth and remains dependent only on IV lipids. Approximately 2 years after her initial presentation, she has underwent successful ileostomy takedown and reanastomosis. Her fibrinogen and platelets have remained normal and her angiopoietin-2 levels have normalized with a treatment regimen consisting of steroids, sirolimus, and vincristine.

## Learning points

Kasabach–Merritt phenomenon is an extremely rare coagulopathy that can present in infancy and be indicative of the presence of a spectrum of complex vascular anomalies such as KHE or KLA. When taking care of a young, small, critically ill infant, large clinical teams must remain open to multiple diagnostic considerations and evolve rapidly even as the patient’s status and the emerging objective findings evolve.

## Data Availability

The original contributions presented in the study are included in the article/Supplementary Material, and further inquiries can be directed to the corresponding author.

## References

[1] Lewis D Vaidya R. Kasabach-Merritt syndrome. [Updated 2023 Jul 17]. In: StatPearls [Internet]. Treasure Island, FL: StatPearls Publishing (2025). Available online at: https://www.ncbi.nlm.nih.gov/books/NBK519053/30085595

[2] Kelly M. Kasabach-Merritt phenomenon. Pediatr Clin N Am. (2010) 57:1085–9. 10.1016/j.pcl.2010.07.006 20888459

[3] Rodriguez V Lee A Witman PM Anderson PA. Kasabach-Merritt phenomenon: case series and retrospective review of the mayo clinic experience. J Pediatr Hematol Oncol. (2009) 31:522–6. 10.1097/MPH.0b013e3181a71830 19564750

[4] Fernandes VM Fargo JH Saini S Guerrera MF Marcus L Luchtman-Jones L Kaposiform lymphangiomatosis: unifying features of a heterogeneous disorder. Pediatr Blood Cancer. (2015) 62:901–4. 10.1002/pbc.25278 25307772

[5] Croteau SE Liang MG Kozakewich HP Alomari AI Fishman SJ Mulliken JB Kaposiform hemangioendothelioma: atypical features and risks of Kasabach-Merritt phenomenon in 107 referrals. J Pediatr. (2013) 162:142–7. 10.1016/j.jpeds.2012.06.044 22871490 PMC3494787

[6] Karnsakul W Ingviya T Seaberg E Laengvejkal P Imteyaz H Vasilescu A Ascites in children: a single-center experience of 27 years. J Pediatr Gastroenterol Nutr. (2017) 64:83–8. 10.1097/MPG.0000000000001209 27050055

[7] Birch JM Blair V. The epidemiology of infant cancers. Br J Cancer. (1992) Suppl 18:S2–4. PMC2149659 1503921

[8] Noris M Caprioli J Bresin E Mossali C Pianetti G Gamba S Relative role of genetic complement abnormalities in sporadic and familial aHUS and their impact on clinical phenotype. Clin J Am Soc Nephrol. (2010) 5:1844–59. 10.2215/CJN.02210310 20595690 PMC2974386

[9] Le Cras TD Mobberley-Schuman PS Broering M Fei L Trenor CC Adams DM. Angiopoietins as serum biomarkers for lymphatic anomalies. Angiogenesis. (2017) 20:163–73. 10.1007/s10456-016-9537-2 27990590

[10] Blatt J Stavas J Moats-Staats B Woosley J Morrell DS. Treatment of childhood kaposiform hemangioendothelioma with sirolimus. Pediatr Blood Cancer. (2010) 55:1396–8. 10.1002/pbc.22766 20730884

[11] Ji Y Chen S Xia C Zhou J Jiang X Xu X Chronic lymphedema in patients with kaposiform hemangioendothelioma: incidence, clinical features, risk factors and management. Orphanet J Rare Dis. (2020) 15:313. 10.1186/s13023-020-01595-2 33160383 PMC7648422

[12] Ji Y Chen S Yang K Xia C Li L. Kaposiform hemangioendothelioma: current knowledge and future perspectives. Orphanet J Rare Dis. (2020) 15:39. 10.1186/s13023-020-1320-1 32014025 PMC6998257

[13] Drolet BA Trenor CC Brandão LR Chiu YE Chun RH Dasgupta R Consensus-derived practice standards plan for complicated kaposiform hemangioendothelioma. J Pediatr. (2013) 163:285–91. 10.1016/j.jpeds.2013.03.080 23796341

[14] Shirazi F Cohen C Fried L Arbiser JL. Mammalian target of rapamycin (mTOR) is activated in cutaneous vascular malformations *in vivo*. Lymphat Res Biol. (2007) 5:233–6. 10.1089/lrb.2007.1012 18370913

[15] Adams DM Trenor CC Hammill AM Vinks AA Patel MN Chaudry G Efficacy and safety of sirolimus in the treatment of complicated vascular anomalies. Pediatrics. (2016) 137:e20153257. 10.1542/peds.2015-3257 26783326 PMC4732362

[16] Ji Y Chen S Peng S Xia C Li L. Kaposiform lymphangiomatosis and kaposiform hemangioendothelioma: similarities and differences. Orphanet J Rare Dis. (2019) 14:165. 10.1186/s13023-019-1147-9 31277673 PMC6612206

[17] Wang Z Li K Yao W Dong K Xiao X Zheng S. Successful treatment of kaposiform lymphangiomatosis with sirolimus. Pediatr Blood Cancer. (2015) 62:1291–3. 10.1002/pbc.25422 25598153

[18] Zukerberg LR Nickoloff BJ Weiss SW. Kaposiform hemangioendothelioma of infancy and childhood. An aggressive neoplasm associated with Kasabach-Merritt syndrome and lymphangiomatosis. Am J Surg Pathol. (1993) 17:321–8. 10.1097/00000478-199304000-00001 8494101

[19] Perez-Atayde AR Debelenko L Al-Ibraheemi A Eng W Ruiz-Gutierrez M O’Hare M Kaposiform lymphangiomatosis: pathologic aspects in 43 patients. Am J Surg Pathol. (2022) 46:963–76. 10.1097/PAS.0000000000001898 35385405 PMC12880111

[20] Ji Y Yang K Peng S Chen S Xiang B Xu Z Kaposiform haemangioendothelioma: clinical features, complications and risk factors for Kasabach-Merritt phenomenon. Br J Dermatol. (2018) 179:457–63. 10.1111/bjd.16601 29603128 PMC11032113

[21] Liu Q Jiang L Wu D Kan Y Fu F Zhang D Clinicopathological features of kaposiform hemangioendothelioma. Int J Clin Exp Pathol. (2015) 8:13711–8. 26722599 PMC4680544

[22] Barclay SF Inman KW Luks VL McIntyre JB Al-Ibraheemi A Church AJ A somatic activating NRAS variant associated with kaposiform lymphangiomatosis. Genet Med. (2019) 21:1517–24. 10.1038/s41436-018-0390-0 30542204 PMC6565516

